# The Administration of Iodide of Potassium in Cases with Obstructed Bronchi

**Published:** 1899-05-06

**Authors:** 


THE ADMINISTRATION OF IODIDE OF POTASSIUM IN CASES WITH OBSTRUCTED BRONCHI.

It is not often that the administration of iodide of potassium in syphilitic gumma does harm; hence, in addition to the interest attached to the cases of syphilitic stenosis of the bronchi exhibited at a recent meeting of the Clinical Society by Dr. Rolleston and Dr. Cyril Ogle, there is an added interest in the practical point raised as to the administration of the iodide, for this drug seemed in these cases to have done harm. It had increased the secretion in the obstructed bronchial tubes, and this secretion accumulating and undergoing changes due to decomposition, set up septic bronchitis, broncho-pneumonia, and fever from septic absorption. To avoid this *contretemps* it was recommended that full doses of belladonna should be combined with the iodide in such cases, so as to prevent the secretion of mucus from the bronchial mucous membrane.

